# The Fate of Orally Ingested Microplastics During Cooperative Brood Care in Two Social Hymenoptera Species

**DOI:** 10.1002/ece3.74006

**Published:** 2026-07-13

**Authors:** Gwen Kühn, Max V. R. Döring, Jona von Wedel, Sven Ritschar, Annalena Ter‐Heide, Valerie Dittmann, Lotta Steinbrenner, Christian Laforsch, Heike Feldhaar

**Affiliations:** ^1^ Animal Population Ecology, Animal Ecology I, Bayreuth Center for Ecology and Environmental Research (BayCEER) University of Bayreuth Bayreuth Germany; ^2^ Animal Ecology I, Bayreuth Center for Ecology and Environmental Research (BayCEER) University of Bayreuth Bayreuth Germany

**Keywords:** carry‐over effects, infrabuccal pouch, regurgitation, social stomach, trophallaxis

## Abstract

Anthropogenic pollutants contribute to insect decline in terrestrial ecosystems. Microplastics (MP), a major pollutant, are already present in all ecosystems and expected to further accumulate. In social insects, negative effects of MP could not only manifest on an individual but also colony level due to cooperative brood care. However, food is transferred differently during cooperative brood care among social Hymenoptera species. Therefore, it can also vary whether particles are passed on with food. Consequently, depending on the strategy of food transfer, the different life stages of a species could be affected by MP to varying degrees. To better assess interspecific differences, we comparatively investigated the fate of polystyrene MP particles during cooperative brood care in colonies of the Japanese carpenter ant ( *Camponotus japonicus* ) and the buff‐tailed bumblebee ( *Bombus terrestris* ). Here we show that both ant and bumblebee workers ingested MP particles with their food. However, we only found MP particles in the digestive system of bumblebee workers and larvae, but not in ant larvae and only occasionally in workers. This is likely due to the infrabuccal pocket (IBP), only present in the ants, that effectively prevents the transfer of particulate matter within colonies during cooperative brood care. In contrast, the unobstructed transfer of MP from bumblebee workers to larvae may entail negative effects on larvae or carry‐over effects during development. Thus, negative colony‐level effects of MP particles on social Hymenoptera may be exacerbated in those species that lack an IBP.

## Graphical abstract

Microplastics (MP) pose a growing health risk to social Hymenoptera, with colony‐level risks depending on brood care strategies. During cooperative brood care, in the buff‐tailed bumblebee, MP is transferred from workers to larvae, but in the Japanese carpenter ant, the infrabuccal pocket prevents such transfer. Thus, MP may have greater colony‐level impacts in species lacking this protective structure.

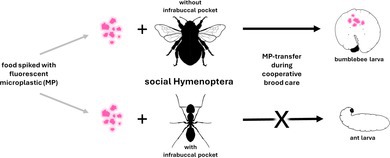

## 1 Introduction

Global biodiversity loss including the massive decline of terrestrial insect abundance and diversity (Dirzo et al. 2014; Wagner et al. 2021) is threatening ecosystem functionality and consequently human well‐being (Díaz et al. 2006). Aside from habitat destruction and intensification of land use, anthropogenic pollutants are another major driver for this decline (Sánchez‐Bayo and Wyckhuys 2019). Therefore, anthropogenic pollutants have gained increased attention over the last years. One of the most prominent examples of anthropogenic pollution is plastic pollution, as it is already exceeding planetary boundaries (Villarrubia‐Gómez et al. 2024). Improperly disposed plastic debris can break down in the environment (Koelmans et al. 2022; Manzoor et al. 2022), for example through mechanical stress (Browne et al. 2007), exposure to UV light (Menzel et al. 2022), ozone (Allen et al. 2003), or through microbial biodegradation (Rohrbach et al. 2024). The amount of the resulting (secondary) microplastics (MP), defined as plastic particles smaller than 1000 μm (International Standard ISO 24187 2023), is increasing in the environment. This increase parallels the rise in global plastic production (Plastics Europe 2025) and the accumulation of improperly disposed plastic waste in the environment (Geyer et al. 2017).

Terrestrial ecosystems are particularly affected by MP pollution, since most of the plastic waste is discarded within continental environments (Horton et al. 2017). Additionally, MP is translocated within terrestrial ecosystems after its generation, for instance via biosolid application (Nizzetto et al. 2016; Weithmann et al. 2018), atmospheric deposition (Sridharan et al. 2021; Kernchen et al. 2024), or rainfall‐induced surface runoffs (Han et al. 2022). Therefore, terrestrial organisms even in unspoilt natural areas will likely face some form of MP pollution, which can negatively affect their health.

Lethal and sublethal effects of MP have already been observed in insects. Orally ingested MP was shown to enhance the susceptibility to pathogen infestation in the honeybees *Apis cerana* and *Apis mellifera* (Deng et al. 2021) and interfere with memory in bumblebees (Cappa et al. 2025) and honeybees (Pasquini et al. 2024). It can additionally decrease the diversity of gut microbiota and alter the expression of genes related to immunity, antioxidants, and detoxification in the guts of *A. mellifera* (Wang et al. 2021, 2022). Exposure to or ingestion of MP can also negatively affect growth and body size of insects (Shah et al. 2023), as observed for the body length and head capsule of a non‐biting midge, the Rice Bloodworm *Chironomus tepperi* (Ziajahromi et al. 2018).

Although individual‐level effects of MP ingestion are increasingly well documented across insects, little is known about the redistribution and retention of orally ingested MP within colonies of social insects. Social insects such as some bees and wasps, ants, and termites are characterised by overlapping generations within the colony, division of reproductive labour, and cooperative brood care (Wilson 1971). Workers forage for food to feed themselves and their nestmates. If they are confronted with MP during foraging, it can accidentally be ingested or attach to their body surface. For instance, forager bees are efficient samplers of airborne pollutants (Negri et al. 2015; Pellecchia and Negri 2018) including MP (Edo et al. 2021; Wang et al. 2022). It has already been observed that workers transport MP back to their colony (Edo et al. 2021), and it is assumed it can be distributed to nestmate workers as well as brood, potentially resulting in increased colony‐level negative effects (Feldhaar and Otti 2020; Li et al. 2024). The first evidence supporting this assumption has been found in honeybees that incorporated ingested MP and other particulate pollutants in their honey and wax combs and transferred it to the brood and non‐foraging nestmates (Papa et al. 2021; Alma et al. 2023). However, feeding strategies during cooperative brood care can differ markedly between species of social Hymenoptera, which may critically influence MP transfer within colonies and the resulting health consequences.

The Japanese carpenter ant *Camponotus japonicus* is widely distributed in East Asia (Dhadwal and Bharti 2021) and mainly feeds on arthropod prey and honeydew (Wu and Wang 1995 fide Wang et al. 2019). In this species, the larvae are fed by nursing workers directly by mouth‐to‐mouth food transfer, so‐called trophallaxis (Meurville and LeBoeuf 2021). Furthermore, these ants have an infrabuccal pocket (IBP), an invagination of the hypopharynx in the oral cavity, which is a filtering device that can filter out particulate matter such as MP from food before it enters the crop (Eisner and Happ 1962; Richter and Economo 2023; Le Hen et al. 2024). Due to its filtering capabilities, it could therefore protect individual workers from potential adverse effects of MP ingestion. As the IBP is anterior to the crop, it may additionally prevent the trophic transfer of MP to brood when crop content is regurgitated during trophallaxis. However, it is unclear whether MP are completely filtered out or if some particles can pass the IBP and reach the crop and/or midgut. In bees, another superfamily of social Hymenoptera, no equivalent to the IBP is known and ingested MP could thus be passed on from workers to larvae during cooperative brood care. One example for a species without IBP is the buff‐tailed bumblebee *Bombus terrestris* . As a generalist widespread in Europe, *B. terrestris* pollinates numerous plant species (Velthuis and van Doorn 2006). In contrast to *C. japonicus* , *B. terrestris* workers do not engage in trophallaxis, but they regurgitate a mixture of nectar and pollen (and secretions from the hypopharyngeal gland) on the larval body surface, from where the larvae feed on it (Goulson 2010).

Since food transfer during cooperative brood care varies among social Hymenoptera, this can lead to MP affecting the different life stages in the colony to varying degrees. To enable a better assessment of health consequences of MP pollution for social insect colonies, we investigated the fate of MP within colonies during cooperative brood care and potential transfer from foraging workers to brood. For the comparison of MP transfer within colonies between *C. japonicus* and *B. terrestris* , we analysed the IBP and digestive system of ant workers, the digestive systems of bumblebee workers, and brood (larvae and pupae) of both species for the presence of MP. We hypothesise that ant workers can filter out almost all MP using their IBP, resulting in an MP‐filled IBP and mostly MP‐free crop and gut. The filtered sugar water from their crop is transferred to their brood; therefore, we expect larvae and pupae to be MP‐free. In contrast, we hypothesise that bumblebee workers cannot filter out MP from their food. We therefore expect to find MP in their gut system and due to a subsequent transfer, also in their brood.

## 2 Materials and Methods

### 2.1 Production and Characterisation of Fluorescent Labelled Microplastics

**FIGURE A1:**
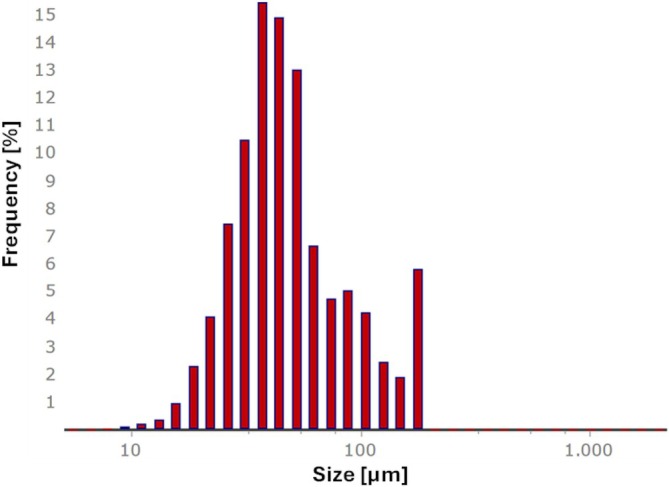
Size distribution of PS microplastic particles. The size is given on the logarithmic x‐axis in micrometres and the frequency is given on the y‐axis in percent.

We used irregular‐shaped fluorescent polystyrene (PS) particles labelled with rhodamine‐b (*λ*_max_ = 544 nm) for feeding trials, as PS is one of the most abundant MPs found in the environment (Wagner et al. 2014). Fluorescent plastic granules (Magic Pyramid Brücher & Partner KG, Frechen, Germany) were milled (centrifugal mill ZM 200, RETSCH GmbH, Haan, Germany; rotor: 24Z; sieve: distance sieve 120 μm) and subsequently sieved to achieve the particle size class we worked with. Fifty percent of the particles had a diameter (*d*_50_) smaller than 40.5 μm (*d*_10_ = 26.86 μm, *d*_90_ = 127.1 μm; Figure A1). The size distribution was determined by a Microtrac Sync particle analyser (Microtrac RETSCH GmbH, Haan, Germany).

### 2.2 Animal Husbandry

The ants and bumblebees were kept in a climate chamber at a constant temperature of 26°C and 70% humidity under an inverted 12:12 h dark: light cycle. Two *C. japonicus* colonies (ANTSTORE, Berlin, Germany) were fed twice per week with honey water (2:1 ratio of water and honey) and cockroaches (*Blaptica dubia*) ad libitum. Six *B. terrestris* colonies were ordered from Biobest (Westerlo, Belgium). The colonies were kept in their delivery boxes, and each was fed three times per week with approximately 10 g of pollen (Imkerpur, Osnabrück, Germany) and sugar water (1:1 ratio of water to Apiinvert [Südzucker AG, Mannheim, Germany]) ad libitum.

### 2.3 Choice of Microplastic Concentrations

Here we focussed on observing the potential transfer of particles within colonies of social insects and not on detecting potential negative effects of MP‐exposure. Consequently, we used fluorescence labelled MP and the concentrations we used were significantly higher than MP concentrations that currently occur in natural environments and rather chosen to facilitate the recovery of the particles in the insect body. For instance, with 0.4% w/v (MP in feeding suspension), we provided the bumblebees with MP‐concentrations that are approximately a thousandfold higher than those currently found in nature (e.g., 4.5 mg per kg in dry weight (0.00045% w/w) of agricultural soils, Büks and Kaupenjohann 2020). Since ants possess the IBP as a powerful filtering device, we chose even higher concentrations of MP (2% w/v). While these MP concentrations significantly exceed concentrations that are considered environmentally relevant today, MP accumulation in the environment is predicted to increase exponentially, and the concentrations used in our study could be realistically reached over the course of the next century (Meizoso‐Regueira et al. 2024).

### 2.4 Microplastics Exposure of *C. japonicus*

For the ant MP‐exposure experiment, we used four plastic boxes (Polypropylene, 20 cm × 14.5 cm × 9 cm) where the bottom was filled with a 1 cm thick layer of plaster. In each box, we placed a 15 mL tube filled with deionised water and closed with a cotton ball to provide constant moisture. We carefully transferred 60 minor workers and 35 larvae into each box to establish microcolonies. Two microcolonies were randomly assigned as a control (sugar water only [1:1 ratio of water to Apiinvert]) and two for the MP exposure (sugar water mixed with 2% w/v MP and 0.02% v/v Tween‐80: SIGMA‐ALDRICH CHEMIE GmbH, Taufkirchen, Germany). The use of controls was important to check for autofluorescence of body structures of the insects and ensure clear differentiation of MP particles from such. The solutions for both treatments were freshly prepared each week and stored at 4°C. Each day, the solutions were vortexed for 10 s, after which the ants were fed with 400 μL of their respective solution.

We continued the feeding trials until at least ten larvae per box had pupated, which took four and nine days in the two control microcolonies and seven and 15 days for the MP treated microcolonies. In one MP treated microcolony, we prematurely ended the feeding trial after 15 days with only eight pupae, since the workers started to feed on their own larvae. Subsequently, the workers and remaining larvae were euthanized with ethyl acetate vapour and stored in 4% paraformaldehyde (PFA) in PBS (v/v; pH = 7.4; MORPHISTO GmbH, Offenbach am Rhein, Germany) at 4°C until further analysis. The pupae were kept in a petri dish in the climate chamber under the above‐mentioned conditions until they reached the P7 stage where abdominal and antennal pigmentation had finished (Ishii et al. 2005). Afterwards, they too were euthanized with ethyl acetate and stored in 4% v/v PFA (in PBS) at 4°C until further analysis of the potential presence of MP in their tissues.

### 2.5 Microplastics Exposure of *B. terrestris* Colonies

**FIGURE A2:**
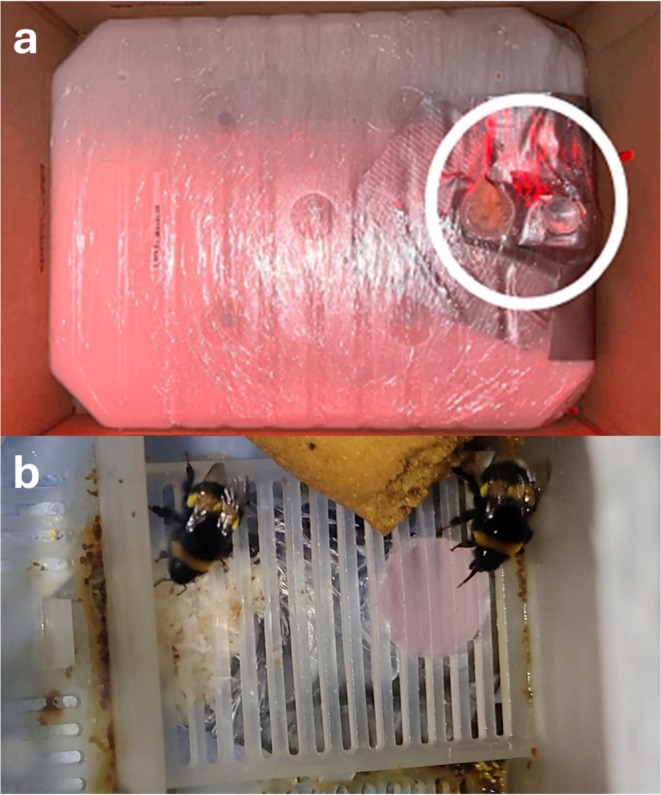
Experimental setup for bumblebees, with (a) the modified sugar water tank with the reservoir taped next to the drinking wick (circled in white) and (b) two bumblebee workers simultaneously drinking from the wick (left) and the reservoir (right, providing fluorescent microplastics suspension).

**FIGURE A3:**
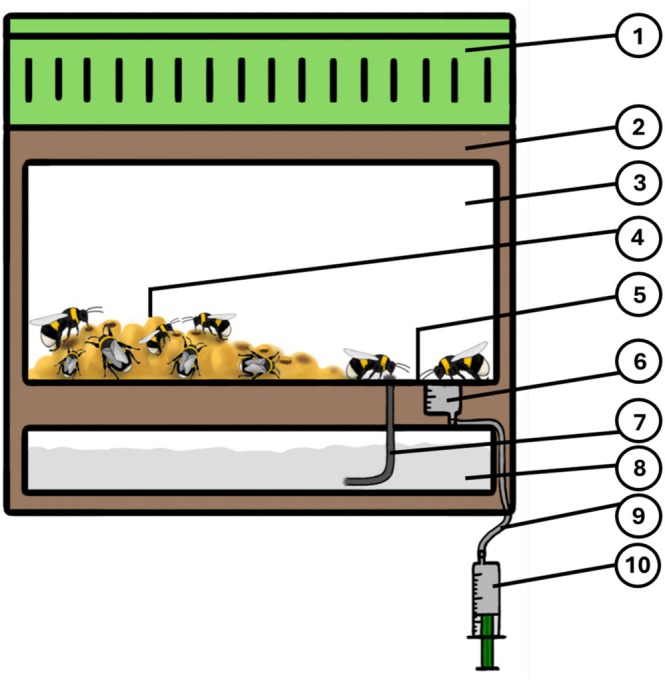
Schematic drawing of the bumblebee colony set up for microplastics exposure. The set up consists of the (1) roof of colony box with air holes, (2) delivery box, (3) colony box, (4) hive area, (5) drinking area with grid‐bottom, (6) reservoir for providing the treatment solution/suspension made from a piece of tube of 10‐mL syringes (Omnifix, B. Braun SE), (7) drinking wick for sugar water from the sugar water tank, (8) sugar water tank, (9) tube (PVC, inner diameter: 4 mm, wall thickness: 1 mm, ZEUS GmbH & Co. KG, Soltau, Germany) to connect the reservoir with the syringe (for refilling the reservoir without disturbing the colony), and (10) a 20‐mL syringe (Omnifix, B. Braun SE) containing the treatment solution/suspension.

We used six queenright *B. terrestris* colonies to obtain three colony‐replicates per treatment. The colonies were assigned randomly to one of the two treatments: control and MP. The control was supplied with pure sugar water (1:1 ratio of water to Apiinvert) and the MP treatment with sugar water, 0.02% v/v Tween 80, and 0.4% w/v MP particles. These treatment solutions/suspensions were provided from 20‐mL syringes in addition to the ad libitum sugar water supply from a tank beneath the colonies. Therefore, we modified the feeding areas of the colonies and placed reservoirs, connected to the syringes providing the treatment solutions/suspensions, underneath the bottom grid. Thus, we ensured that the workers could reach the spiked food with their mouthparts only to prevent bumblebee workers from spreading the particles throughout the colony via body contact (for further details see pictures in Figure A2 and schematic set‐up with description in Figure A3).

To prevent growth of mould or bacteria, the syringes providing the treatment solutions/suspensions were changed three times per week and the colonies were fed with approximately 10 g of pollen (organic flower pollen: DE‐ÖKO‐037, Imper Pur; Osnabrück, Germany). We refreshed the sugar water from the tanks once a week.

Bumblebee colonies were fed over a period of 4 weeks to ensure the development of pupae and the emergence of imagines that, during their larval stages, had potentially been fed with MP by their nestmates. Then the colonies were anaesthetised with CO_2_ by placing the colony boxes on dry ice in a closed styrofoam container for several minutes. Subsequently, larvae, pupae, and workers were collected from the colonies for further analyses, and the remaining colonies were euthanized in the freezer at −20°C overnight.

### 2.6 Detection of Microplastic Particles in *C. japonicus* and *B. terrestris*

To detect fluorescent labelled PS particles on the body surface of *C. japonicus* , we haphazardly selected 20 workers and all larvae and pupae from each microcolony. We investigated them using a Leica M205 FCA fluorescence stereo microscope (Leica Microsystems GmbH, Wetzlar, Germany) equipped with a Leica DBC6200 camera and the Leica Application Suite X software (version 3.8.2.27713). We used a red fluorescence filter (excitation: 540–580 nm, emission: 593–667 nm) for the fluorescence images. If fluorescent labelled PS particles were found on an individual, it was carefully submerged in acetone for 10 s to dissolve the PS and therefore eliminate the fluorescence. The procedure was repeated until we observed no more fluorescence on the body surface. This ensured that any fluorescent particles detected within the digestive system of the workers could only have come from MP that was ingested.

We checked the digestive system including the IBP of workers, larvae (that do not possess an IBP), and the entire body of pupae for the presence of MP. In worker ants we analysed the IBP and the rest of the digestive system separately (described as ‘IBP’ and ‘digestive system’ hereafter). The dissection was done in a blinded manner. For our investigations we used two different methods: half of the individuals investigated per microcolony were dissected under a stereo microscope while the other half was subjected to a tissue clearing protocol described by Ritschar et al. (2022). Tissue clearing enables in situ detection of MP particles without prior dissection.

For the tissue clearing process of the ants an adaptation of the CUBIC (Clear Unobstructed, Brain Imaging, Cocktails and computational analysis) protocol (Susaki et al. 2015) was used. We produced the required solutions CUBIC‐1 and CUBIC‐2 as follows: For 500 g of the CUBIC‐1 solution 125 g urea (Grüssing GmbH, Filsum, Germany) and 156 g of 80% w/w quadrol (N,N,N′,N′‐Tetrakis(2‐hydroxypropyl)ethylenediamine, Sigma‐Aldrich, Merck KGaA, Darmstadt, Germany), dissolved in milli‐Q H_2_O were dissolved in 144 mL milli‐Q H_2_O on a hot plate at 80°C. After all ingredients were dissolved, 75 g Triton‐X‐100 (Thermo Scientific, Life Technologies GmbH, Darmstadt, Germany) were added under continuous stirring. For 500 g of the CUBIC‐2 solution 125 g urea (Grüssing GmbH, Filsum, Germany) and 250 g sucrose (Carl Roth GmbH + Co. KG, Karlsruhe, Germany) were dissolved in 75 g of milli‐Q H_2_O on a hot plate at 60°C–65°C. Then the solution was cooled to room temperature and 50 g of triethanolamine (SIGMA‐ALDRICH CHEMIE GmbH, Taufkirchen, Germany) were added under continuous stirring. For 50% concentrations (½ CUBIC‐1 and ½ CUBIC 2), the solutions were diluted with PBS. The CUBIC clearing protocol was then performed as follows: The individuals, that were stored in the 4% PFA in PBS were rinsed in PBS (pH = 7.4) three times for 5 min each. Afterwards, they were placed in embedding cassettes (Simport Scientific Inc., Boleil, Canada) in sample containers (Faust Lab Science GmbH, Klettgau, Germany) with each separate container holding one developmental stage (e.g., adult, larvae, or pupae) of one treatment. The loaded containers were then filled with 0.03% H_2_O_2_ (Fisher Scientific, Loughborough, United Kingdom) in 98% technical EtOH and placed on a shaker at 37°C with renewal of the solution every other day for depigmentation (for around 1 week). When no further bubble formation occurred, and hence no further bleaching of the individuals could be observed in the current H_2_O_2_ solution, the concentration was first increased to 3% (for around 2 weeks) and subsequently, when also here no further bleaching was visible, to 5% (for around 5 weeks). The H_2_O_2_ concentration had to be increased slowly to avoid excessive oxygen production and hence to reduce air bubble entrapment in the specimens as much as possible. When the depigmentation procedure was completed, removal of the lipids followed. Therefore, the ants were placed in ½ CUBIC‐1 (1:1 PBS and CUBIC‐1) overnight (also on a shaker at 37°C). After the ½ CUBIC‐1 solution, the samples were submerged in CUBIC‐1 until the ants were transparent (around 3 weeks). For adjustment of the refractive index, the samples were placed in ½ CUBIC‐2 (1:1 PBS and CUBIC‐2) overnight on a shaker and at room temperature (22°C). After the ½ CUBIC‐2 solution the specimens were placed in CUBIC‐2 (also on a shaker at room temperature) for another 4 days. Then they were screened for fluorescent MP using the Leica M205 FCA fluorescence stereomicroscope (Leica Microsystems GmbH, Wetzlar, Germany) equipped with a Leica DBC6200 camera and the Leica Application Suite X software (version 3.9.0.28093). For fluorescence images we used a red fluorescence filter (excitation: 540–580 nm, emission: 593–667 nm).

Similarly, two sets of individuals were analysed for presence of MP in *B. terrestris* , one for dissection and one for tissue clearing. For dissection, 15 workers, 15 larvae, and 9 pupae were haphazardly selected per colony and stored frozen until examination. The examinations were carried out blinded, using unique random 9‐digit IDs, to rule out any possible bias. All specimens were checked for MP on the body surface using the Leica M205 FCA fluorescence stereo microscope before dissection. In preliminary tests we found that especially the larvae and pupae were very brittle once dipped into acetone, which complicated dissection. Therefore, if MP was found on the body surface, we instead removed the MP with 70% EtOH until the body surface was MP‐free. Only then were the digestive systems dissected from workers and larvae, while the pupae were crushed and analysed for MP. The other set of individuals (one worker, one larva, and five pupae per colony, from 6 colonies) was prepared for tissue clearing by conservation in 4% PFA (in PBS) following the protocol of Ritschar et al. (2022) for *B. terrestris* . Workers, furthermore, were previously carefully shaved using a scalpel. If specimens were damaged during handling, they were excluded from the analysis.

The tissue clearing process of the bumblebees was similar to the clearing of the ants with minor changes only. At first, the specimens deriving from 4% PFA were rinsed in 1× PBS thrice for 5 min. For the subsequent steps adult and larvae individuals were placed separately in 20 and 10 mL syringes (Omnifix and Injekt syringes, B. Braun SE, Melsungen, Germany), respectively, whereas always five pupae (deriving from the same colony) were pooled in a 20 mL syringe for further processing. After washing the samples in 1× PBS they were also depigmented using the same rising H_2_O_2_ concentrations as for the ants with exchange of the solution every other day. Here the duration also varied between the different life stages as follows: All three live stages (adults, larvae and pupae) spent around 2 weeks in 0.03% H_2_O_2_. The adults then spent around 1 week in 3% H_2_O_2_, whereas larvae and pupa were in this solution for around 6 weeks. While the larvae and pupa were already bleached after this step, the adults passed another 5 weeks in 5% H_2_O_2_. When the depigmentation procedure was completed, the adults and pupae were transferred from the syringes to centrifuge tubes (15 and 50 mL) for further handling. Then the lipids were also removed by placing the bumblebees in ½ CUBIC‐1 (around 1 week for adults and pupae and around one and a half weeks for larvae) and subsequently in CUBIC‐1 (both on a shaker at 37°C) until they were transparent (after around 3 weeks for adults and pupae and around 8 weeks for larvae). As the depigmentation and clearing steps took longer for the bumblebees than for the ants, the bumblebee individuals were more fragile. Therefore, to avoid tissue damage, they did not go through the ½ CUBIC‐2 and CUBIC‐2 steps but instead were immediately screened for fluorescent MP as described for the ants.

### 2.7 Statistical Analysis

All statistical analyses were conducted using R 4.4.1 (R Core Team 2024). The barplots were created and arranged using the packages *ggplot2* (ver. 3.5.2; Wickham 2016) and *ggpubr* (ver. 0.6.0; Kassambara 2023) respectively. In monogynous colonies of social insects like *B. terrestris* and *C. japonicus* all offspring are closely related to each other. Therefore, the number of colonies represents the true number of biological replicates, while individuals deriving from the same colony are not independent of each other.

To best accommodate the data structure, we opted for generalised linear models (GLMs), although we are aware of the limitations posed by the small number of true replicates. For the GLMs, we used a binomial error distribution and logit link function to account for the binary presence‐absence data. We used the number of individuals with and without MP in their digestive system (or inner body), aggregated per colony, as response variable for the models (*dplyr* package, ver. 1.1.4, Wickham et al. 2023). For each species and life stage we fitted an individual model. We introduced conservative dummy variables (i.e., variables reducing the actual difference between two treatments without altering the number of observations), to avoid estimation problems that arise when groups exclusively comprise of zero entries (no MP present; e.g., the controls). Since in ant larvae and bumblebee pupae no MP was detected, we fitted no models for these life stages.

## 3 Results

### 3.1 Ingestion of MP by *C. japonicus*

**FIGURE 1:**
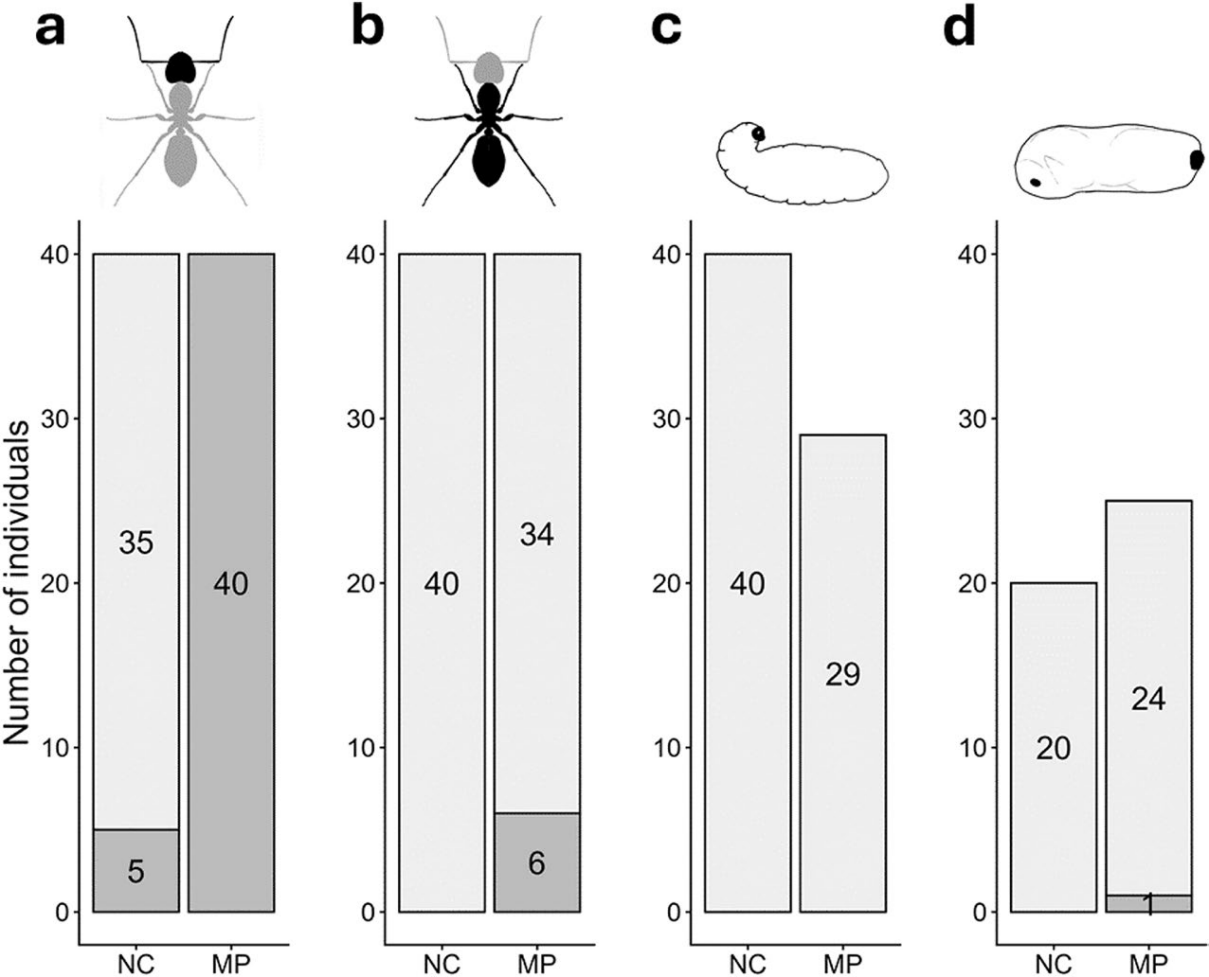
Results for microplastics (MP) detection in ant workers, larvae and pupae. Number of individuals with (dark grey) and without (light grey) fluorescent MP detected (a) in the IBP of ant workers, (b) digestive system of ant workers, (c) digestive system of ant larvae or (d) the inner body of ant pupae from microcolonies fed with control solution (NC = negative control; *N* = 2 colonies) or fluorescent MP suspension (*N* = 2 colonies).

We observed that workers from all microcolonies were feeding on their supplied sugar water and performed trophallaxis with their nestmates. Five control workers each had a single fluorescent particle in their IBP, probably due to cross contamination. However, we found significantly more workers of the MP treatment with fluorescent particles in their IBP (40 out of 40 workers) compared to the control (5 out of 40 workers; ANOVA, *χ* ^2^ = 79.5, df = 1, *p* < 2 × 10^−16^; Figure 1).

**FIGURE 2:**
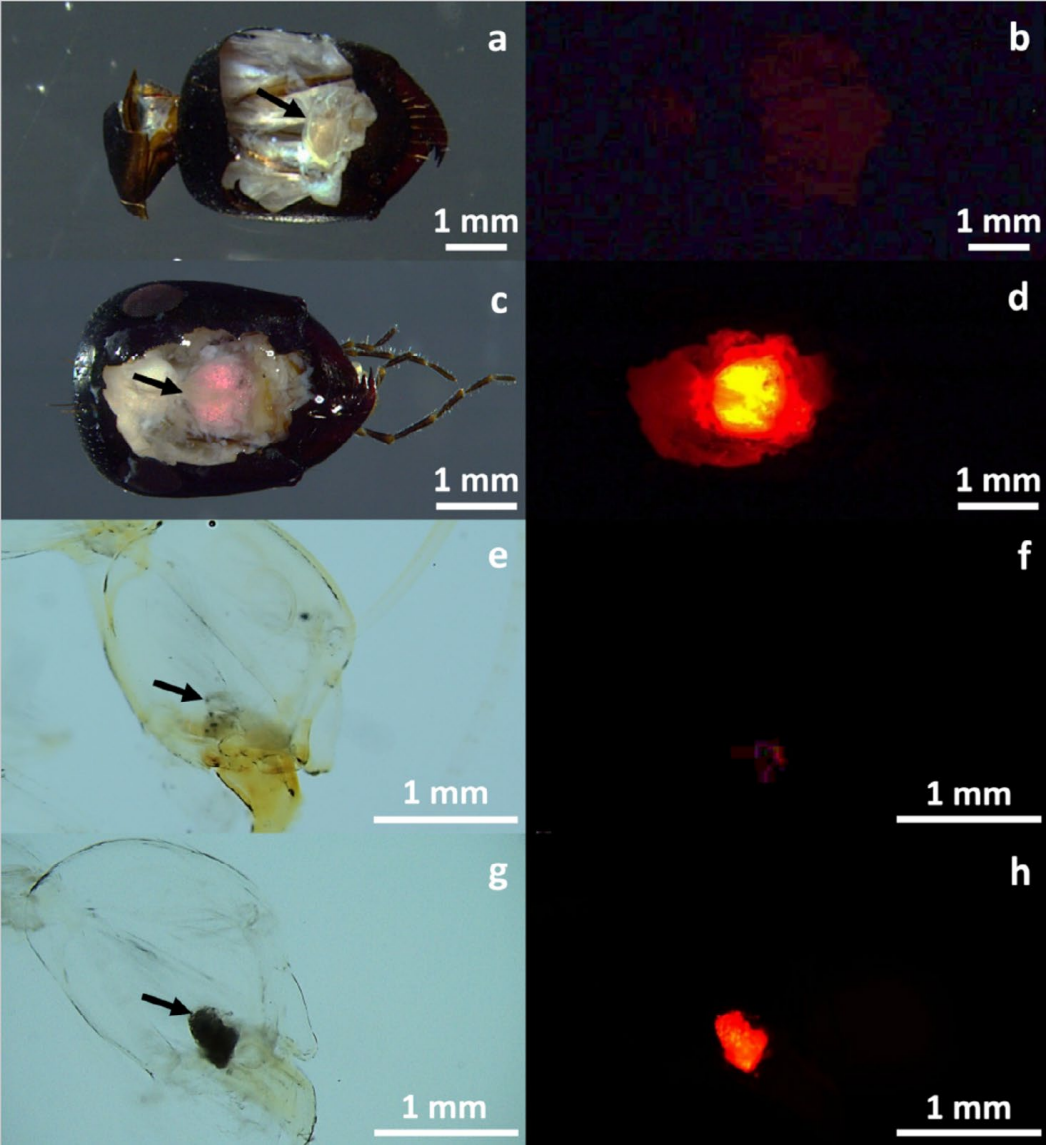
Exemplary head sections of the *Camponotus japonicus* workers illustrating the function of the infrabuccal pocket (IBP) as a filtering device. Images show workers fed only with control solution (a, b, e, f) or fluorescent microplastics (MP) suspension (c, d, g, h). Black arrows point at the IBPs. (a) and (c): Dorsal view of the opened head using light microscopy; (b) and (d): Dorsal view of the opened head using fluorescence microscopy; (e) and (g): Lateral view of a tissue cleared head using light microscopy; (f) and (h): Lateral view of a tissue cleared head using fluorescence microscopy. To improve the visibility of non‐MP structures on the fluorescence images, the brightness of image (b) was increased by 65% and the brightness of image (f) was increased by 95%.

**FIGURE A4:**
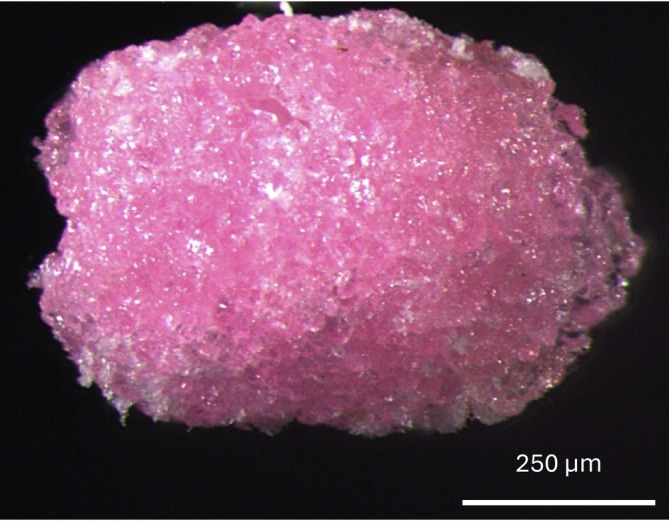
Example of an MP pellet from the infrabuccal pocket of a *Camponotus japonicus* worker.

We found multiple MP pellets (see Figure A4) and pellet fractions on refuse piles and elsewhere within the nest boxes in the MP treated microcolonies and none in the control microcolonies. Six of 40 workers from the MP treatment had fluorescent particles in their digestive system following the IBP, but none of the control workers (ANOVA, *χ* ^2^ = 4.3, df = 1, *p* = 0.038; Figure 1). The IBP was often completely filled with MP (Figure 2) and appeared in a pink colour using light microscopy (Figure 2c) or dark after the tissue clearing (Figure 2g), and exhibited strong fluorescence (Figure 2d,h). No larvae of both treatments contained any MP, and only one pupa (from the MP treatment; ANOVA, *χ* ^2^ = 0.026, df = 1, *p* = 0.87; Figure 1) had a single fluorescent particle in its oral cavity.

### 3.2 Ingestion of MP by *B. terrestris* Workers

**FIGURE 3:**
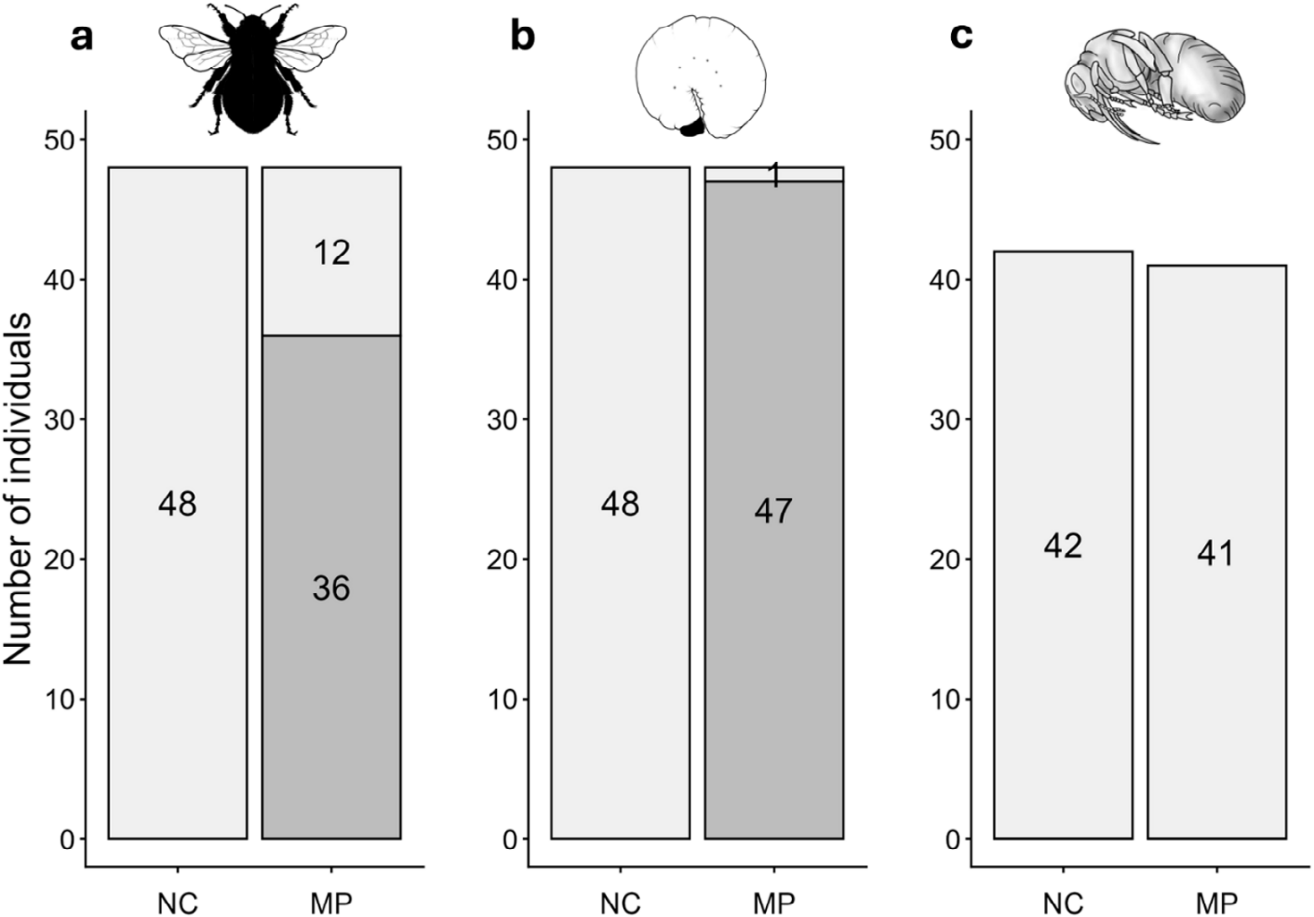
Results for microplastics (MP) detection in bumblebee workers, larvae, and pupae. Number of individuals with (dark grey) and without (light grey) MP particles detected (a) in bumblebee workers, (b) bumblebee larvae and (c) bumblebee pupae from colonies fed with control solution (NC = negative control, *N* = 3 colonies) or fluorescent MP suspension (*N* = 3 colonies).

**FIGURE A5:**
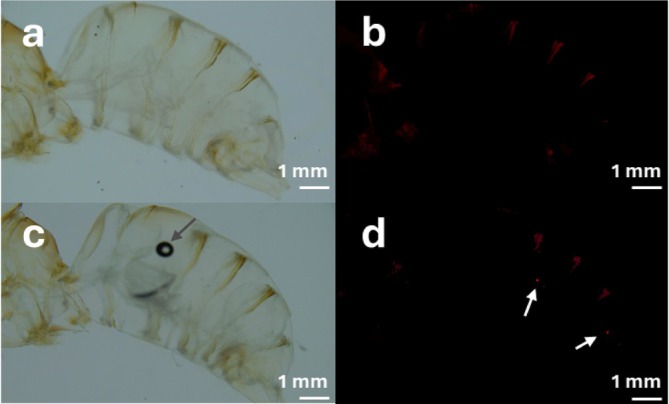
Tissue cleared abdomen of *Bombus terrestris* workers fed with control solution (a, b) or fluorescent microplastics (MP) suspension (c, d). From each specimen a picture was taken with light (a, c) and fluorescent (b, d) microscopy, to detect fluorescent MP particles. For better visibility, fluorescent MP particles are indicated by white arrows (d). The dark spot visible in picture c is an air bubble trapped in the tissue, that occasionally cannot be prevented. The air bubble is indicated by a grey arrow. Less bright and less sharply defined fluorescence, which can also be seen on the image without fluorescent MP particles (b), is caused by autofluorescence of the individuals' own body structures. To improve the visibility of non‐MP structures on the fluorescence images, the brightness of image (b) was increased by 90% and the brightness of image (d) was increased by 91%.

We observed several *B. terrestris* workers to frequently feed on the MP suspensions in addition to the sugar water (personal observation; Figure A2b). The detection frequency of MP in the digestive systems of bumblebee workers differed significantly between treatments (ANOVA, *χ* ^2^ = 60.6, df = 1, *p* = 6.9 × 10^−15^). In 36 out of 48 workers (=75%) from colonies provided with the MP suspension, we found MP in their digestive system (Figure 3a; Figure A5c,d). None of the digestive systems of the 48 workers from the control colonies contained any MP (Figure 3a; Figure A5a,b).

### 3.3 Transmission of MP From *B. terrestris* Workers to Offspring

**FIGURE A6:**
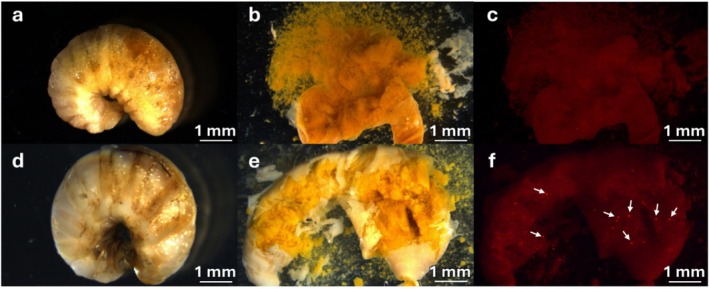
*Bombus terrestris* larva from colonies fed with control solution (a–c) or fluorescent microplastics (MP) suspension (d–f). Pictures were taken from the intact body (a, d) and from the opened digestive system (b, c, e, f). Pictures from the opened digestive systems were taken with light (b, e) and fluorescent (c, f) microscopy, to detect fluorescent MP particles. For better visibility, particles are indicated by white arrows. Less bright and less sharply defined fluorescence, which can also be seen on the image without fluorescent MP particles, is caused by autofluorescence of the individuals' own body structures. To improve the visibility of non‐MP structures on the fluorescence images, the brightness of images (c) and (f) was increased by 70%, respectively.

The detection frequency of MP particles in the digestive systems of bumblebee larvae was significantly different between treatments (ANOVA, *χ* ^2^ = 105.6, df = 1, *p* < 2 × 10^−16^). We found MP particles in the digestive systems of 47 out of 48 larvae (=98%) from colonies provided with the MP suspension (Figure 3b; Figure A6d–f). None of the larvae from control (*n* = 48) colonies had any MP particles in their digestive system (Figure 3b; Figure A6a–c). Over all colonies and treatments, we did not find any MP particles in the inner body of any of the bumblebee pupae analysed (Figure 3c; control: *n* = 42; MP: *n* = 41).

## 4 Discussion

MP particles were orally ingested by workers with feeding suspensions by both social Hymenoptera species we investigated, the ant *C. japonicus* , and the bumblebee *B. terrestris* . The transfer of MP from workers to offspring differed strongly between *C. japonicus* and *B. terrestris* . While MP was not transferred to larvae in *C. japonicus* it was found in almost all larvae in *B. terrestris* . MP was found only in a single pupa in *C. japonicus* and in none of *B. terrestris* . We must point out that our results are based on a limited number of truly independent replicates (i.e., colonies). We nevertheless consider this to only have a minor impact on the validity of our results.

### 4.1 MP Uptake in Workers

The IBP is a filtering device of some ant species such as *C. japonicus* , which can filter out particulate matter such as MP from food before it enters the crop (Eisner and Happ 1962; Richter and Economo 2023; Le Hen et al. 2024). Previous studies have shown that ants, including *C. japonicus* (e.g., Wei et al. 2025), avoid food spiked with MP, suggesting that they are able to detect MP contamination (Le Hen et al. 2024; Wei et al. 2025). However, in our study, all ant workers from the MP treatment harboured MP particles in the IBP. This was likely due to the experimental design where workers had no choice, since they were exclusively provided with MP spiked food. In spite of the high concentrations of MP used in our study, only 15% of these workers also had MP in their digestive system, suggesting that some particles passed the IBP and reached the crop and/or midgut. Workers of *C. pennsylvanicus* were able to completely filter out corundum particles > 150 μm, whereas particles smaller than 100 μm were found in the crop (Eisner and Happ 1962). Workers of the red imported fire ant *Solenopsis invicta* were even able to filter out almost all particles ≥ 0.88 μm (Glancey et al. 1981). This suggests that the IBP can only effectively filter out particles above a certain species‐dependent size. While most MP was filtered out in our case, some of the presumably smallest particles were still able to pass through (with 50% of PS particles being smaller than 40.5 μm). In addition, we found that the workers regurgitated the collected MP as compact MP pellets, cleaning the IBP from MP and restoring its filter capacity. Therefore, the IBP likely decreases the risk of adverse effects of MP taken up with food since most particles are filtered out before reaching the digestive system.

In contrast, bumblebee workers do not have a structure equivalent to the IBP. Accordingly, we frequently found MP particles in their digestive system. Unlike ants (Wang et al. 2019; Le Hen et al. 2024), bumblebees are most likely not able to filter out particulate pollution from feeding suspensions before it enters their digestive system. Therefore, bumblebee workers may have a health disadvantage compared to ants due to the lack of the IBP when exposed to MPs.

### 4.2 MP Transfer From Workers to Larvae

Since the IBP is located anterior to the crop, it may prevent the trophic transfer of MP to brood provided from crop content during trophallaxis. In our study, we could not find any MP in the digestive systems of ant larvae, even though all workers fed with MP also contained it in their IBP and in some workers, it had reached their gut. This suggests that ant workers do not (or very rarely) transmit MP to their brood during trophallaxis, which protects ant larvae from the direct negative effects of MP particles.

In contrast, we found MP in all but one of the investigated larval digestive systems of bumblebees. Since larvae are fed by workers, the MP must be ingested by workers before it is regurgitated with liquid food for feeding larvae. This is particularly interesting in the context of a recent study on metal avoidance during cooperative brood care in bumblebees (Gekière et al. 2025). In this study, bumblebees appear to recognise metal pollution in food. In the absence of brood, workers ingested metal polluted food. By contrast, in the presence of brood, workers avoided spiked food and thus did not feed it to their brood. Despite having the chance to do so in our experimental set up, not all bumblebee workers avoided ingesting and passing on MP spiked food during cooperative brood care. This suggests that during oral ingestion, MP may not be recognised as a pollutant by bumblebee workers, emphasising the particular hazard potential of MP pollution in the environment.

A transfer of MP from workers to larvae during cooperative brood care has also been observed in *A. mellifera* by Alma et al. (2023). Together with our results, this suggests that the transfer of particulate pollutants from workers to brood during cooperative brood care could be common among bees due to their presumed inability to filter out most particles from liquid food. The consequences of MP transfer from bumblebee workers to larvae can be versatile. Larvae could experience adverse effects of MP particles such as higher mortality (Al‐Jaibachi et al. 2019; Muhammad et al. 2021; Buteler et al. 2022) or sublethal effects such as reduced body weight (Al Naggar et al. 2023), alterations in the expression of genes related to immunity and/or detoxification (Muhammad et al. 2021; Wang et al. 2021), as well as increased susceptibility towards other environmental contaminants (Cho et al. 2020). Furthermore, pollution of food with indigestible particles may result in food dilution and subsequent negative effects on growth or development of larvae, as previously observed for other organisms (Amariei et al. 2022; Bucci et al. 2024). Detrimental effects could be carried over into the pupal and/or adult stage and result in smaller, weaker or otherwise negatively affected workers. Such carry‐over effects could then also negatively impact colony health and functioning (Choe and Rust 2008; Siviter et al. 2018; Schläppi et al. 2021; Stuligross and Williams 2021).

### 4.3 MP Presence in Pupae

Since we did not find any MP in ant larvae, it is unsurprising that only a single ant pupa contained MP particles. Apart from the IBP in workers, ants as holometabolous insects purge their digestive system as last instar larvae, which is visible as the meconium after cocoon formation in those species that form a cocoon (Gotoh et al. 2023). This potentially liberates the pupa from previously ingested MP. Larvae of the paper wasp *Polistes satan* were able to completely remove MP from their gut system by defecating prior to pupating, as no MP was found in the developed adults (Rodrigues de Souza et al. 2023). Likewise, the larvae of *B. terrestris* gradually stop feeding but continue defecation until their digestive system is completely emptied to prepare for the prepupal stage (Pereboom 2001). This is in line with our results that none of the pupae analysed contained MP.

Remaining MP could cause severe problems during the metamorphosis and negatively affect the future worker's health. For midges, the transfer of MP (2 μm in diameter) from larval to adult stages has been shown before (Al‐Jaibachi et al. 2018, 2019; Setyorini et al. 2021), with MP particles being retained in the digestive system (Setyorini et al. 2021) or possibly also in Malphigian tubules (Al‐Jaibachi et al. 2018, 2019) during metamorphosis. Further, drone fly larvae ( *Eristalis tenax* ) that were reared in MP‐spiked water with 5000 polyamide fragments/mL contained MP in their gut as adults. Pupal and imagines' weight was reduced by 33% and 60%, respectively, when compared to individuals developing in uncontaminated water (Abdulla et al. 2025). In bumblebees, however, we did not detect MP inside the bodies of pupae. Therefore, emptying the digestive system seems to be an effective measure to remove particulate pollutants from the bumblebee body before metamorphosis.

In this context, we want to emphasise that this mechanism is effective for the particulate matter only. However, there is evidence that toxic substances can be transferred from MP into the tissues of organisms (Browne et al. 2013; Avio et al. 2015; Ašmonaitė et al. 2018; Batel et al. 2018; Lu et al. 2018; Qu et al. 2019, 2020). The health effects of such leaching events from MPs into the tissues of terrestrial insects have hardly been researched so far, but they may pose a major health risk also for social insects.

## 5 Conclusion

Here, we comparatively investigated the transfer of MP as a common particulate environmental pollutant from workers to larvae during cooperative brood care in two social Hymenoptera species. Workers of both the ant *C. japonicus* as well as the bumblebee *B. terrestris* ingested MP particles. However, in contrast to bumblebees, the ants did not transfer MP to their offspring, likely because they are filtered out by the IBP that ants but not bumblebees possess. This protective effect of the IBP, however, is effective only against particulate pollutants. Soluble chemicals such as additives that may potentially leach from the MP particles could still enter the digestive system and from there other tissues. Bumblebee workers fed on the MP‐spiked food suspension and transferred ingested MP to colony offspring. Bumblebee workers could therefore have a limited ability to recognise MP pollution of food sources. The different results for these two social Hymenoptera species emphasise the importance of the feeding strategy for the resulting health consequences.

Overall, our results underscore the vulnerability to the effects of particulate pollutants of social Hymenoptera that do not possess an IBP. Due to the unfiltered transfer of particulate pollutants from workers to colony offspring during cooperative brood care, not only workers but whole colonies may be affected.

## Author Contributions

**Gwen Kühn:** conceptualization (equal), data curation (equal), formal analysis (equal), investigation (equal), methodology (equal), visualization (equal), writing – original draft (equal), writing – review and editing (equal). **Max V. R. Döring:** conceptualization (equal), data curation (equal), formal analysis (equal), investigation (equal), methodology (equal), visualization (equal), writing – original draft (equal), writing – review and editing (equal). **Jona von Wedel:** investigation (equal), methodology (equal), visualization (equal), writing – original draft (equal), writing – review and editing (equal). **Sven Ritschar:** investigation (equal), methodology (equal), visualization (equal), writing – original draft (equal), writing – review and editing (equal). **Annalena Ter‐Heide:** investigation (equal), methodology (equal), visualization (equal). **Valerie Dittmann:** investigation (equal), methodology (equal), visualization (equal). **Lotta Steinbrenner:** investigation (equal), methodology (equal), visualization (equal). **Christian Laforsch:** conceptualization (equal), funding acquisition (equal), resources (equal), supervision (equal), writing – review and editing (equal). **Heike Feldhaar:** conceptualization (equal), funding acquisition (equal), resources (equal), supervision (equal), writing – review and editing (equal).

## Funding

This work was supported by Deutsche Forschungsgemeinschaft (DFG, German Research Foundation; SFB 1357 Mikroplastik—Project Number 391977956), the Studienstiftung des deutschen Volkes (doctoral scholarship Gwen Kühn), and the Marianne‐Plehn program (scholarship Gwen Kühn).

## Conflicts of Interest

The authors declare no conflicts of interest.

## Data Availability

Data and code are available online at Mendeley Data under: Büchner, Gwen; Döring, Max V. R.; Schmitt, Jona; Ritschar, Sven; Ter‐Heide, Annalena; Dittmann, Valerie; Steinbrenner, Lotta; Laforsch, Christian; Feldhaar, Heike (2026), “Trophic_Transfer_of_Microplastics_in_Social_Hymenoptera”, Mendeley Data, V5, https://doi.org/10.17632/d3y42pvx5x.5 (https://data.mendeley.com/datasets/d3y42pvx5x/5).
